# Using family planning service statistics to inform model-based estimates of modern contraceptive prevalence

**DOI:** 10.1371/journal.pone.0258304

**Published:** 2021-10-29

**Authors:** Niamh Cahill, Emily Sonneveldt, Priya Emmart, Jessica Williamson, Robinson Mbu, Airy Barrière Fodjo Yetgang, Isaac Dambula, Gizela Azambuja, Alda Antonio Mahumane Govo, Binod Joshi, Sayinzoga Felix, Clarisse Makashaka, Victor Ndaruhutse, Joel Serucaca, Bernard Madzima, Brighton Muzavazi, Leontine Alkema

**Affiliations:** 1 Department of Mathematics and Statistics, Maynooth University, Maynooth, Kildare, Ireland; 2 Avenir Health, Glastonbury, CN, United States of America; 3 Department of Family Health, Ministry of Public Health, Yaoundé, Cameroon; 4 Central Monitoring and Evaluation Division, Ministry of Health, Lilongwe, Malawi; 5 Child Health Department, Ministry of Health, Maputo, Mozambique; 6 Women’s Health Division, National Directorate of Public Health, Mozambique; 7 Department of Health Services, Ministry of Health and Population, Kathmandu, Nepal; 8 Maternal, Child and Community Health Division, Rwanda Biomedical Centre, Kigali, Rwanda; 9 National AIDS Council, Harare, Zimbabwe; 10 Ministry of Health and Child Care, Harare, Zimbabwe; 11 School of Public Health and Health Sciences, University of Massachusetts Amherst, Amherst, MA, United States of America; University of Salamanca, SPAIN

## Abstract

The annual assessment of Family Planning (FP) indicators, such as the modern contraceptive prevalence rate (mCPR), is a key component of monitoring and evaluating goals of global FP programs and initiatives. To that end, the Family Planning Estimation Model (FPEM) was developed with the aim of producing survey-informed estimates and projections of mCPR and other key FP indictors over time. With large-scale surveys being carried out on average every 3–5 years, data gaps since the most recent survey often exceed one year. As a result, survey-based estimates for the current year from FPEM are often based on projections that carry a larger uncertainty than data informed estimates. In order to bridge recent data gaps we consider the use of a measure, termed Estimated Modern Use (EMU), which has been derived from routinely collected family planning service statistics. However, EMU data come with known limitations, namely measurement errors which result in biases and additional variation with respect to survey-based estimates of mCPR. Here we present a data model for the incorporation of EMU data into FPEM, which accounts for these limitations. Based on known biases, we assume that only changes in EMU can inform FPEM estimates, while also taking inherent variation into account. The addition of this EMU data model to FPEM allows us to provide a secondary data source for informing and reducing uncertainty in current estimates of mCPR. We present model validations using a survey-only model as a baseline comparison and we illustrate the impact of including the EMU data model in FPEM. Results show that the inclusion of EMU data can change point-estimates of mCPR by up to 6.7 percentage points compared to using surveys only. Observed reductions in uncertainty were modest, with the width of uncertainty intervals being reduced by up to 2.7 percentage points.

## 1. Introduction

Monitoring changes in modern contraceptive use provides valuable insight into the impact of a country’s family planning (FP) program [1]. For over 2 decades the family planning community has relied on large national-scale surveys such as the Demographic and Health Survey (DHS) and the Multiple Indicator Cluster Survey (MICS) to track progress in family planning indicators, such as the modern contraceptive prevalence rate (mCPR) and unmet need for contraception [2, 3]. Organizations that are invested in monitoring FP progress require the ability to capture current trends in FP indicators and project them into the future [4]. The United Nations Population Division (UNPD) uses The Family Planning Estimation Model (FPEM [5, 6]) for the purposes of producing annual estimates and projections of FP indicators over time. The Track20 project (www.track20.org), responsible for monitoring progress towards achieving the goals of the Family Planning 2020 (FP2020 [7]) initiative, uses a country-specific implementation of FPEM via a user-friendly web application called the Family Planning Estimation Tool (FPET [8]). This country-specific implementation of FPEM is also available as an R package (*fpemlocal* [9, 10]). The surveys that inform these model-based estimates can have lags of up to five years or longer between subsequent data points (surveys are infrequently carried out every 3–5 years on average). Time lags result in recent estimates that are not data driven.

All countries collect routine service statistics data, ranging from basic logistical information about numbers of family planning services and stock levels of FP commodities to more detailed indicators on the characteristics of family planning clients [11]. These data can be reported as often as on a monthly basis and can therefore allow for more real-time monitoring of FP programs. However, there are challenges with using these data to produce population level FP indicators comparable with the data produced by the large scale-surveys. Potential pitfalls include the lack of standardization of service statistics across countries, reporting errors and failure to capture information from the private sector [11]. With the aim of addressing some of these challenges, Track20 have developed a tool that converts service statistics data into a single metric, Estimated Modern Use (EMU). This “SS to EMU” tool [12] converts different types of service statistics data, including family planning commodities distributed, visits to FP facilities/providers or family planning users into EMU.

FPEM was originally set up with the intention of estimating FP indicators using survey observations only [5]. However, with the introduction of initiatives such as FP2020 and the Track20 monitoring program facilitating the collection and processing of service statistics into EMUs in recent years, the incorporation of information from service statistics into the country-specific implementation of FPEM has been explored [6]. When considering how to use EMU data in conjunction with FPEM, it is important to be aware of, and account for, the inherent biases associated with EMUs. Magnani et al., established that while EMU observations fall short in tracking the actual level of mCPR in a way that is comparable to population-based surveys, they *can* potentially track changes in mCPR [11]. In previous iterations of FPEM (outlined in the appendix of Cahill et al., 2018 [6]), EMU data were used in a data model that linked them to a biased estimate of mCPR [6]. This setup ensured that EMUs did not impact the level of mCPR estimates but could influence the general trend past the most recent survey. The limitations of this approach were that (1) the data model did not directly utilize the relationship between changes in EMUs and changes in mCPR as established in Magnani et al., (2) the magnitude of the variation parameters in the EMU data model were not well understood, and (3) it required two FPEM model runs in order to calculate a bias correction factor.

Based on what we know from the work presented in Magnani et al., as well as our own exploratory analysis, we have updated the EMU data model in FPEM to more directly incorporate the assumption, that despite the EMU level being a biased measurement of mCPR, changes in EMU are unbiased with respect to changes in this indicator. We present this updated data model for EMU data in the context of its use as part of the *fpemlocal* R package [9, 10]. The advantage of the updated approach is that the data model more directly utilizes what we know about the underlying relationship between EMUs and mCPR with respect to biases and variation. In addition, this setup only requires a single FPEM run. We implement the updated EMU data model within the *fpemlocal* R package, and we provide insight into the impact of using EMU data for informing estimates and projections of mCPR for case study countries: Cameroon; Malawi; Mozambique; Nepal; Rwanda and Zimbabwe, where surveys are not available in recent years.

## 2. Data

### 2.1 Family planning indicators

The total contraceptive prevalence rate (CPR) is measured as the percentage of women who report themselves or their partners as currently using at least one contraceptive method of any type (modern or traditional). The modern contraceptive prevalence rate (mCPR) is measured as the percentage of women who report themselves or their partners as currently using a modern contraceptive method. Unmet need for family planning is defined as the percentage of women who want to stop or delay childbearing but who are not currently using any method of contraception to prevent pregnancy. Women of reproductive age (15–49 years) who were currently married or in a union are referred to as married/in-union women of reproductive age (MWRA).

### 2.2 Survey data

Survey observations of contraceptive prevalence and unmet need for family planning used for relevant countries in this paper were obtained from the *fpemlocal* R package [9, 10]. The Track20 data in this package is based on the most recent version of the world contraceptive use database compiled by the United Nations Population Division [13]. Data are obtained from nationally representative household surveys including the Demographic and Health Surveys (DHS), Performance Monitoring and Accountability 2020 surveys, the Multiple Indicator Cluster Surveys, the Reproductive Health Surveys, Contraceptive Prevalence Surveys and World Fertility Surveys.

### 2.3 Estimated Modern Use (EMU) data

Service Statistics are routine family planning program data that are collected in connection with family planning service delivery. For the purposes of this work these include: (1) number of contraceptive commodities distributed to clients and/or facilities such as the number of pill cycles and number of intrauterine devices (shortened to comm. clients/comm. facilities); (2) number of times clients interacted with a provider for contraceptive services (shortened to service visits) and (3) number of current contraceptive users of any method including those who are still using longer acting methods that were received in previous years (shortened to FP Users).

Estimated Modern Use (EMU) is an estimate of modern contraceptive use based on service statistics data and assumptions about to how those data relate to contraceptive usage [14]. In the case of comm.clients (by method), comm.facilities (by method) and service visits (by method), annual counts are converted into annual estimates of numbers of current or active contraceptive users. These estimates are then adjusted to account for under-reporting of the private-sector service statistics. The estimated numbers of adjusted contraceptive users are then divided by the estimated number of women of reproductive age for each country, during each year covered by the service statistics data. This yields approximations of annual mCPR estimates, referred to as EMU. EMUs are obtained by Track20 with the SS to EMU tool [12] to provide EMU data for FP2020-pledging countries [7]. For this study, service statistics data that were available in 2018 from FP2020-pledging countries were processed and compiled. The resulting dataset comprised 213 EMU observations from 27 countries. The data are summarised in Table 1. 13 of the 27 countries listed in Table 1 used their EMU observations for providing the FP2020 report figures in 2018 [15]. These countries are labeled with an Asterix (*).

**Table 1:** A summary of 2018 EMU database. The first column contains the country name with the * indicating if the country used EMU data in the FP2020 2018 report. Column 2 is the number of years of available EMU data, column 3 is the service statistics source type(s) available in that country, column 4 indicates the year of the most recent survey in the 2018 survey database and column 5 is the number of survey observations available up to and including the recent survey year.

Country	No. EMU	Source Type	Recent Survey Year	No, Survey
Afghanistan*	11	comm.clients	2015	10
**Bangladesh**	4	comm.facilities	2014	16
**Benin**	13	comm.clients,service visits	2016	6
Burkina Faso*	4	comm.clients, FP users	2014	8
Cameroon*	4	comm.facilities	2017	8
**Cote d’Ivoire**	3	comm.clients, comm.facilities, FP users	2017	7
**Ghana**	6	comm.clients, FP users	2017	16
**Guinea**	3	comm.clients, service visits	2017	5
**Kenya**	5	service visits	2017	12
**Madagascar**	6	comm.clients	2015	8
Malawi*	6	service visits	2015	9
Mali*	2	comm.clients	2015	7
**Mauritania**	4	comm.facilities, service visits	2015	6
Mozambique*	5	comm.clients	2016	6
**Myanmar**	3	FP users	2016	6
Nepal*	5	comm.clients, comm.facilities, FP users	2016	12
**Niger**	8	comm.clients, FP users	2016	7
Nigeria*	4	comm.clients	2015	11
**Pakistan**	5	comm.clients	2015	19
Rwanda*	5	comm.clients, FP users	2017	8
**Senegal**	3	FP users	2015	12
**Sierra Leone**	6	FP users	2015	6
South Sudan*	3	comm.clients	2014	4
Togo*	11	comm.facilities	2017	6
**Uganda**	4	comm.clients, service visits	2017	11
United Republic of Tanzania*	4	comm.clients, comm.facilities, service visits	2015	8
Zimbabwe*	5	comm.facilities	2016	9

## 3. Methods

FPEM was designed to use survey data related to contraceptive use to inform annual estimates of mCPR, unmet need, and demand for family planning satisfied by modern methods between survey years, beyond the date of the last survey and into the future. FPEM falls under the class of Temporal Models for Multiple Populations [16] (TMMP) and has two main components: a process model and a data model. The process model captures trends over time in the true but unknown family planning outcomes of interest, while the data model specifies how the observed data relates to the outcomes of interest, taking into account different types of error that may be present in different data sources. Model parameters govern the characteristics of the process and data models. In this section, we briefly summarize the FPEM process model for estimating mCPR, the survey data model, and the parameter estimation. Then we introduce the main contribution of this paper, the EMU data model, and discuss how it is parametrized.

### 3.1 Estimating contraceptive prevalence using FPEM

#### Process model for contraceptive prevalence

**Fig 1:**
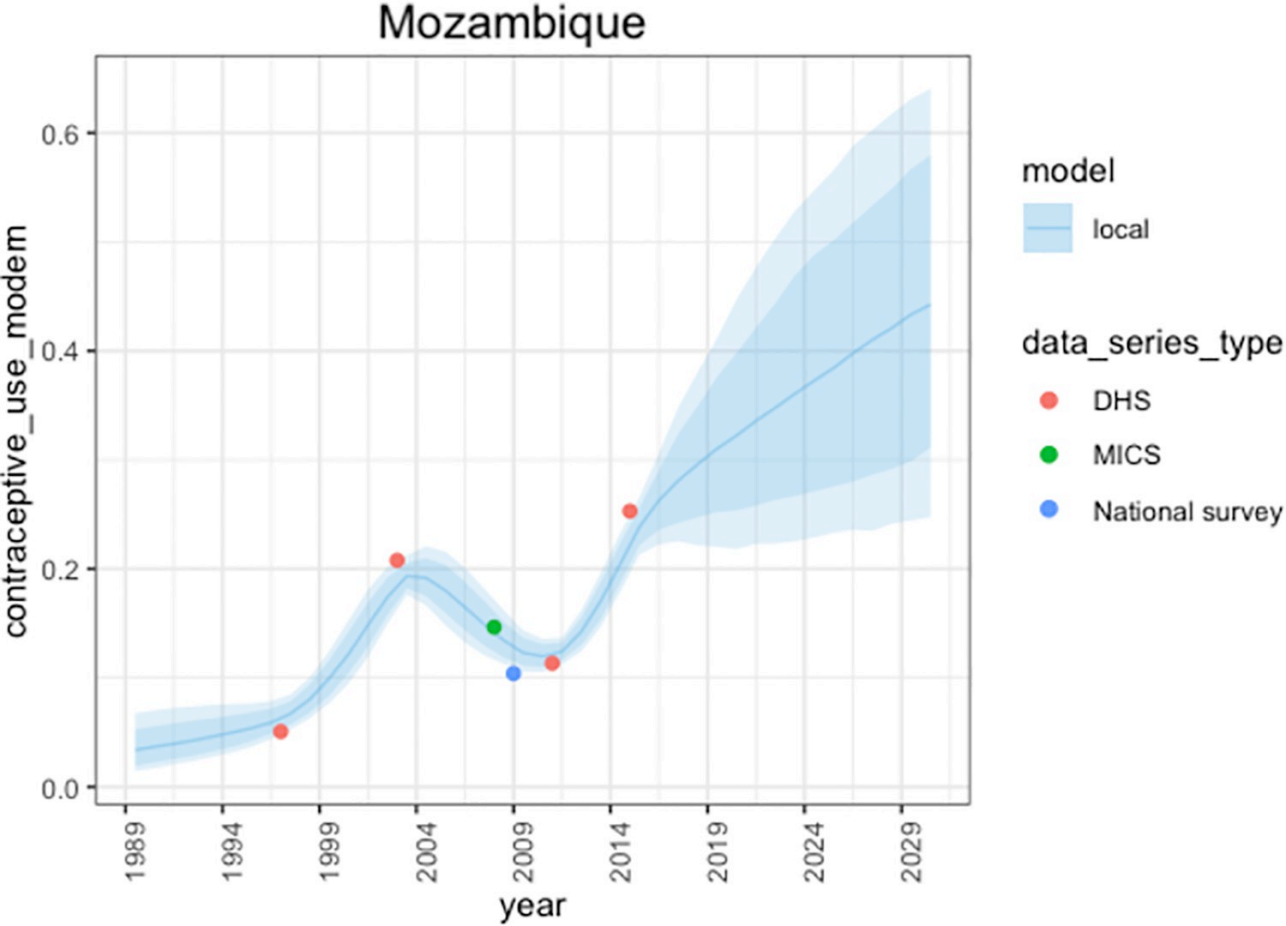
Estimates and projections of Modern Contraceptive Prevalence (mCPR) from the fpemlocal R package for Mozambique. The blue solid line shows the median estimates, the blue shaded regions show the 80% and 95% uncertainty intervals. Survey observations representative of the married-or-in-union women (MW) population are indicated as points and colours represent the survey source.

For each country c, in each year t, FPEM provides estimates of mCPR which we denote *ρ*_*c*,*t*_. This is achieved by modelling total contraceptive prevalence (the aggregate of modern and traditional prevalence) and the ratio of modern to total contraceptive prevalence. Full details of the model specification can be found in the appendix of Cahill et al., 2018 [6]. To summarise, FPEM assumes that the expected rate of change in total CPR at time t, depends on its level at time t-1, as well as the rate of change expected under the assumption of logistic growth. The set up captures the underlying contraceptive prevalence transition from a gradual increase to a more rapid increase and then to a slow down when high levels of prevalence are reached. Time series distortions are added to capture how rates of change in the observed data (i.e., faster/slower rates of change in contraceptive prevalence) deviate from the expected rates of change. The ratio of modern to total prevalence is also specified by assuming logistic growth, with distortions away from expected trends being captured by a time series process. Projections of mCPR are informed by recent changes that have occurred in contraceptive prevalence as well as past experience [5, 6]. An example of survey informed estimates and projections of mCPR from Mozambique, produced by the *fpemlocal* R package are shown in Fig 1.

#### Survey data model

A detailed specification of the survey data model can be found in the appendix of Cahill et al., 2018. To summarise the main idea here, the observed data for each of the modern and traditional contraceptive use categories are linked to the true but unknown proportions (modeled by the process model) through assuming a bivariate normal distribution for the log-ratios of modern and traditional prevalence to no contraceptive use [5]. The relationship between observed and modeled proportions is adjusted for differing data quality and for data that do not pertain to the base population of interest (e.g., data for married women not aged 15–49 years).

#### Parameter estimation

Model parameters are estimated within a Bayesian framework, with prior distributions placed on unknown parameters. The parameters that control the country-specific logistic growth curves are estimated hierarchically, such that estimates are based on the data available in the country of interest, and also the sub-regional, regional, and global experience. In the country-specific implementation of FPEM used by *fpemlocal*, priors on country-specific parameters are obtained from a global implementation of the model [10, 17]. Markov Chain Monte Carlo (MCMC) sampling is implemented to obtain posterior distributions of parameters via the JAGS [18] (Just Another Gibbs Sampler) software within R [19].

### 3.2 Estimating modern contraceptive prevalence using EMUs and FPEM

#### Exploratory data analysis

**Fig 2:**
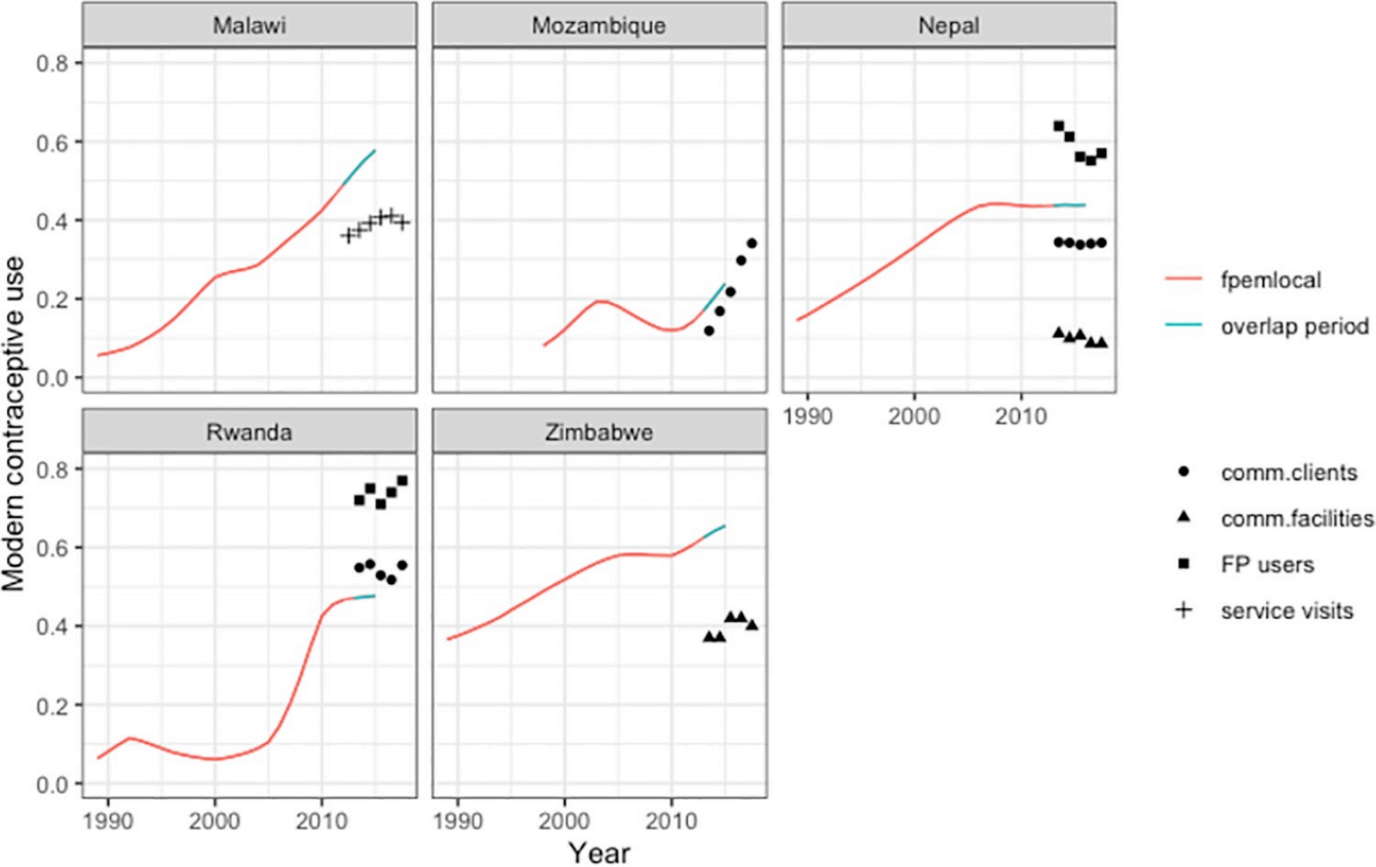
Comparing EMU to model-based mCPR. The symbols represent observations of different EMU data types, depending on availability in the country. The lines are survey informed estimates of mCPR obtained from fpemlocal. The line changes from red to green to indicate the time period where there was an overlap between EMUs and surveys.

Fig 2 illustrates the comparison between *fpemlocal* survey-based estimates of mCPR and EMU data for Malawi, Mozambique, Nepal, Rwanda, and Zimbabwe. EMU data tend to be biased with respect to the true levels of mCPR in a given country, as illustrated in the figure by comparing EMU data to FPEM estimates, which are deemed unbiased estimates of mCPR.

**Fig 3:**
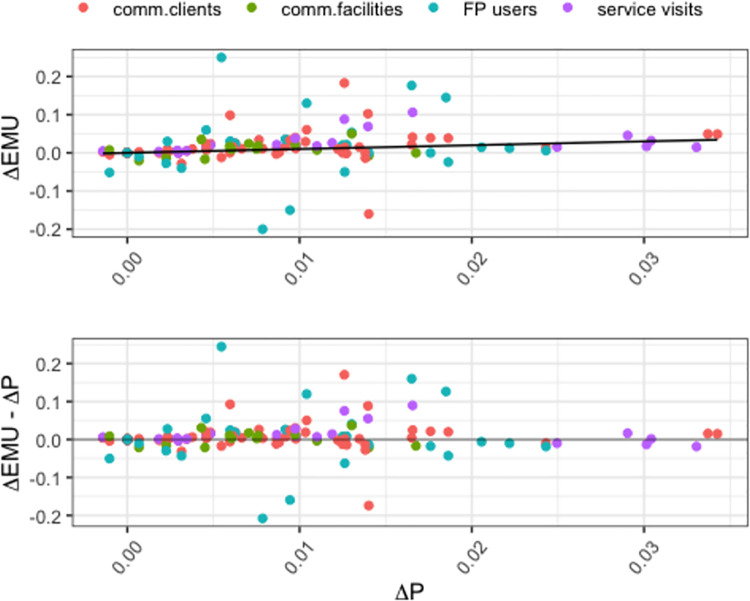
(A) Scatterplots of changes in EMU (ΔEMU) vs changes in survey-informed mCPR estimates from fpemlocal (ΔP). The blue line is the identity line with ΔEMU = ΔP. (B) A scatter plot of (ΔEMU–ΔP) vs ΔP (analogous to a residual vs fit plot). Colours indicate the EMU source type.

Fortunately, changes in EMU observations have been shown to relate to changes in survey-based estimates of mCPR [11]. This is illustrated in Fig 3A, showing the changes in EMU observations (*Δ*EMU) vs changes in survey informed *fpemocal* estimates of mCPR (*Δ*P) by EMU source types. A perfect one to one relationship would result in all points falling on the blue identity line. In this case, the observed changes are scattered around the identity line, with some source types exhibiting more scatter than others. This is further illustrated in Fig 3B which shows that, overall, the bias is centered on 0, but that different EMU data types exhibit different levels of variation around 0.

The data shown in Fig 3 were derived from the 2018 EMU database and fpemlocal estimates of mCPR informed by the Track20 database (filtered to only include surveys in relevant countries prior to 2018). We only used EMU data from country-periods where there was an overlap with a survey, and within those countries, we only used data up until the most recent survey year and nothing beyond that (i.e., the overlap period illustrated in Fig 2). The resulting exploratory EMU data set comprised 150 EMU observations available prior to 2018 from 22 countries, with 11 countries having 1 EMU data type, 8 countries having 2 types and 3 countries having three data types.

#### EMU data model

Let *ρ*_*c*,*t*_ be the true, unknown mCPR in country *c* at any time *t* and *z*_*c*,*t*,*s*_ be an EMU observation of type *s* available at time t in country *c*, where *s* is the service statistics source type (s = 1 (comm. clients), 2 (comm. facilities), 3 (service visits), 4 (FP users)). We propose a data model for EMU data based on observed rates of change. The EMU data model is implemented as:

$$z_{s,c,t}-z_{s,c,t-1}∼N(\rho_{c,t}-\rho_{c,t-1},\sigma_{s}^{2}),$$

with the expected change in EMUs over time in a given country assumed to be equal to the corresponding change in mCPR (*ρ*_*c*,*t*_−*ρ*_*c*,*t*−1_), and the source-type specific variance captured by $\sigma_{s}^{2}$. If and when EMU observations are available, their use comes with the caveat that there must be at least 2 years of available data to calculate a rate of change.

#### Estimating the source-type specific variance

Fig 3B illustrates the magnitude and variance of the change-biases (*Δ*EMU–*Δ*P) across the different EMU data types. We use these data to estimate the source-specific standard deviation (*σ*_*s*_) for the EMU data model. Specifically, for EMU data type *s* with *n*_*s*_ data points, the estimate of *σ*_*s*_, which we will denote ${\hat{\sigma }}_{s}$, is given by

$${\hat{\sigma }}_{s}=\sqrt{\sum_{i=1}^{n_{s}}\frac{{\left({\Delta EMU}_{s,i}–{\Delta P}_{s,i}\right)}^{2}}{n_{s}}}$$

The estimates of *σ*_*s*_ based on this equation are given in Table 2. The variance is largest for EMUs based on FP users, followed by comm.clients, service visits, and finally, comm.facilities.

**Table 2:** Estimates of the standard deviation of differences between changes in EMU observations and changes in survey-informed estimates of mCPR (*Δ*EMU– *Δ*P) across EMU data types.

Type	N	Standard deviation $\left({\hat{\sigma }}_{s}\right)$
**Comm.clients (s = 1)**	52	0.04
**Comm.facilities (s = 2)**	16	0.02
**FP users (s = 3)**	25	0.09
**Service visits (s = 4)**	21	0.03

#### Model fitting

The *fpemlocal* package contains R functions and input data to do model fitting for a single country using FPEM. The primary function *fit_fp_c*, fits the model for a country of interest. If EMU data are provided when using *fpemlocal*, these data will inform estimates and projections of contraceptive use categories through the EMU data model introduced in section 3.2 within the *fpemlocal* modelling architecture. The EMU dataset must be provided as a function argument to fit_fp_c and the dataset must minimally include, a numeric code associated with the country of interest, the EMU observations, the corresponding year and the EMU data type, see “fpemlocal emu” vignette for further details.

### 3.3 Model validation

An out-of-sample validation procedure was set up to look at model performance for the inclusion of the EMU data model in *fpemlocal*. Performance was assessed in terms of predicting the most recent left-out survey observations. The EMU dataset and survey dataset described in the Data section (section 2) provided the training and test data. To obtain the survey training data the Track20 survey database was initially filtered to exclude data after 2018 and subsequently filtered further to exclude the most recent survey observation from each country. The training database was made up of 180 observations of contraceptive prevalence from 24 countries and 117 observations of unmet need from 23 countries between 1968 and 2016. The EMU training data used a single EMU data type per country (as would be the case in practice) and excluded countries where there were no EMU observations after the most recent survey in the survey training dataset. The EMU validation dataset therefore included 128 EMU observations from 24 countries. The test data included the most recent survey observations of contraceptive prevalence that had been excluded from the survey training data.

Performance was measured in terms of coverage (i.e., how often 95% prediction intervals capture the test data points), prediction errors and a prediction interval score [20]. Validation carried out on a survey-only model provided a baseline comparison for the model with EMU data included.

## 4. Results

### 4.1 Model validation

Table 3 presents coverage, mean error, the root mean-squared error (RMSE) and a prediction interval score for the validation exercise. The mean error provides a measure of the average bias in the model predictions. The positive mean errors seen in Table 3 indicate that on average the model tends to slightly underpredict the surveys (by < 2 percentage points). From Table 3 we can see that the inclusion of the EMU data model in *fpemlocal* provides a slight reduction in the mean error for prediction as compared to using the survey-only model predictions. The RMSE provides a summary of the variation in the prediction errors and can be interpreted as a standard deviation. Table 3 shows that both models have an RMSE of about 4 percentage points. The inclusion of the EMU model does not improve the coverage. The prediction interval score rewards for narrow prediction intervals while penalising if an observation is not contained within the interval [20]. Table 3 shows a slight improvement on the prediction interval score when EMUs are included.

**Table 3:** Validation results. Summaries of validation results are shown for 24 countries in the test data set. Coverage is defined as the percentage of time that the true value is captured with the 95% uncertainty intervals. The mean error is the average prediction error. RMSE is the Root Mean Squared Error.

Model	N	Coverage	Mean Error	RMSE	Interval Score
**EMU+Survey**	24	92%	0.014	0.039	0.222
**Survey Only**	24	92%	0.017	0.042	0.269

### 4.2 Impact of including the EMU data model in FPEM

**Fig 4:**
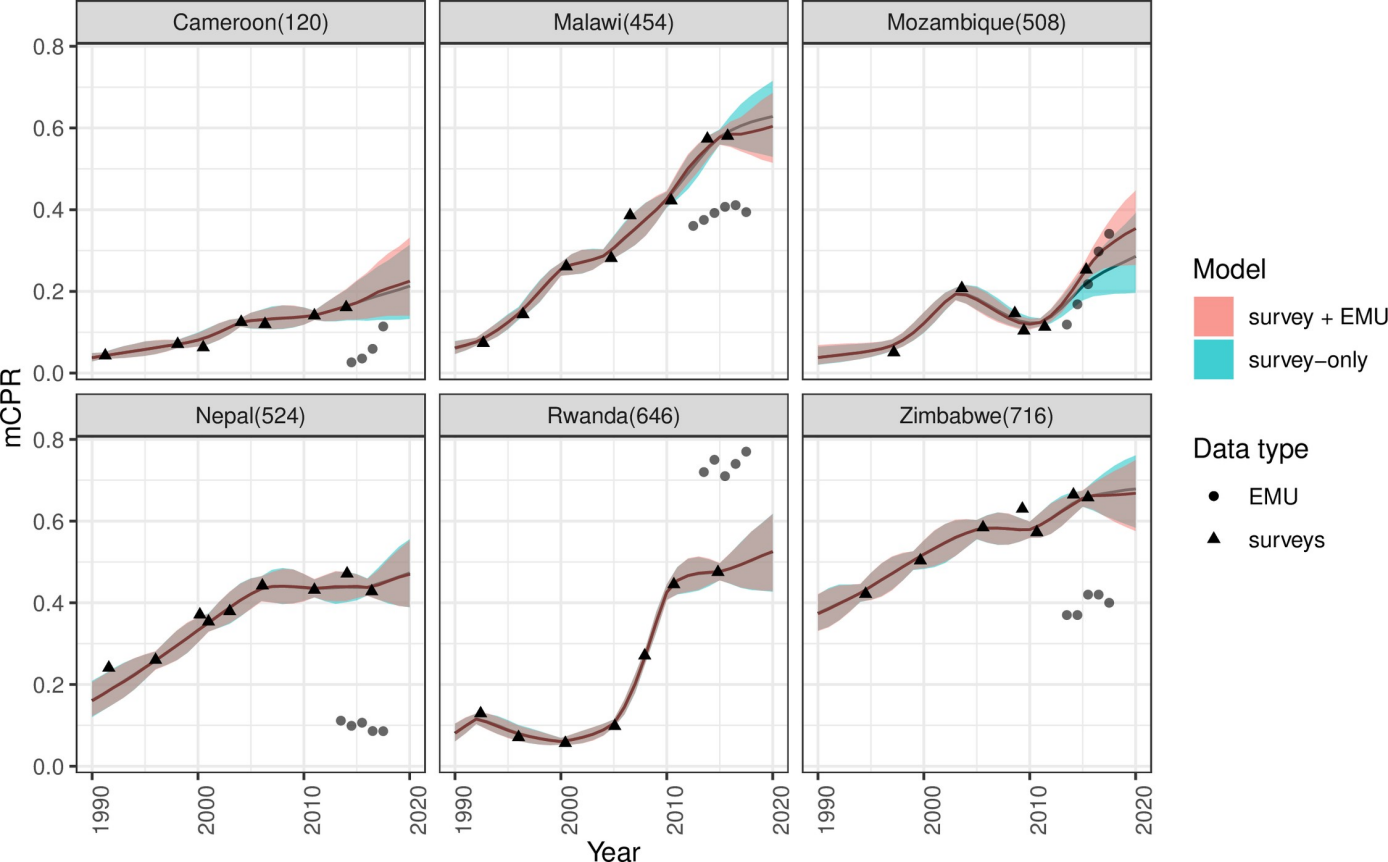
Comparing estimates and projections of mCPR. Solid lines indicate point estimates of mCPR and the shaded regions show the 95% uncertainty intervals. The results in green are from the EMU + survey model and the results in red are from the survey-only model. Shapes of data points distinguish survey observations from EMU.

We assessed the impact that the addition of the EMU data model in FPEM has on the estimates and projections of mCPR. Output was obtained using the *fpemlocal* R package with the contraceptive use database associated with the package and a recent (2019) EMU dataset containing data from 26 FP2020-pledging countries. We compared the mCPR for the year 2020, obtained from *fpemlocal* when using a survey-only model, to the mCPR obtained when using a survey model combined with an EMU data model. We assessed the differences in both mCPR point-estimates and uncertainty, broken down by the 4 EMU source types. Focusing on 6 case study countries, in Fig 4 we can see that where changes between EMUs appear to be larger than changes implied by surveys, there is an uptake in survey+EMU informed mCPR estimates as compared to the survey-only informed estimates (e.g., Cameroon, Mozambique). Conversely, if we see negative changes in EMU, we see a drop in mCPR estimates relative to survey-only informed estimates (e.g., Malawi, Zimbabwe).

#### Impact on point estimates

**Fig 5:**
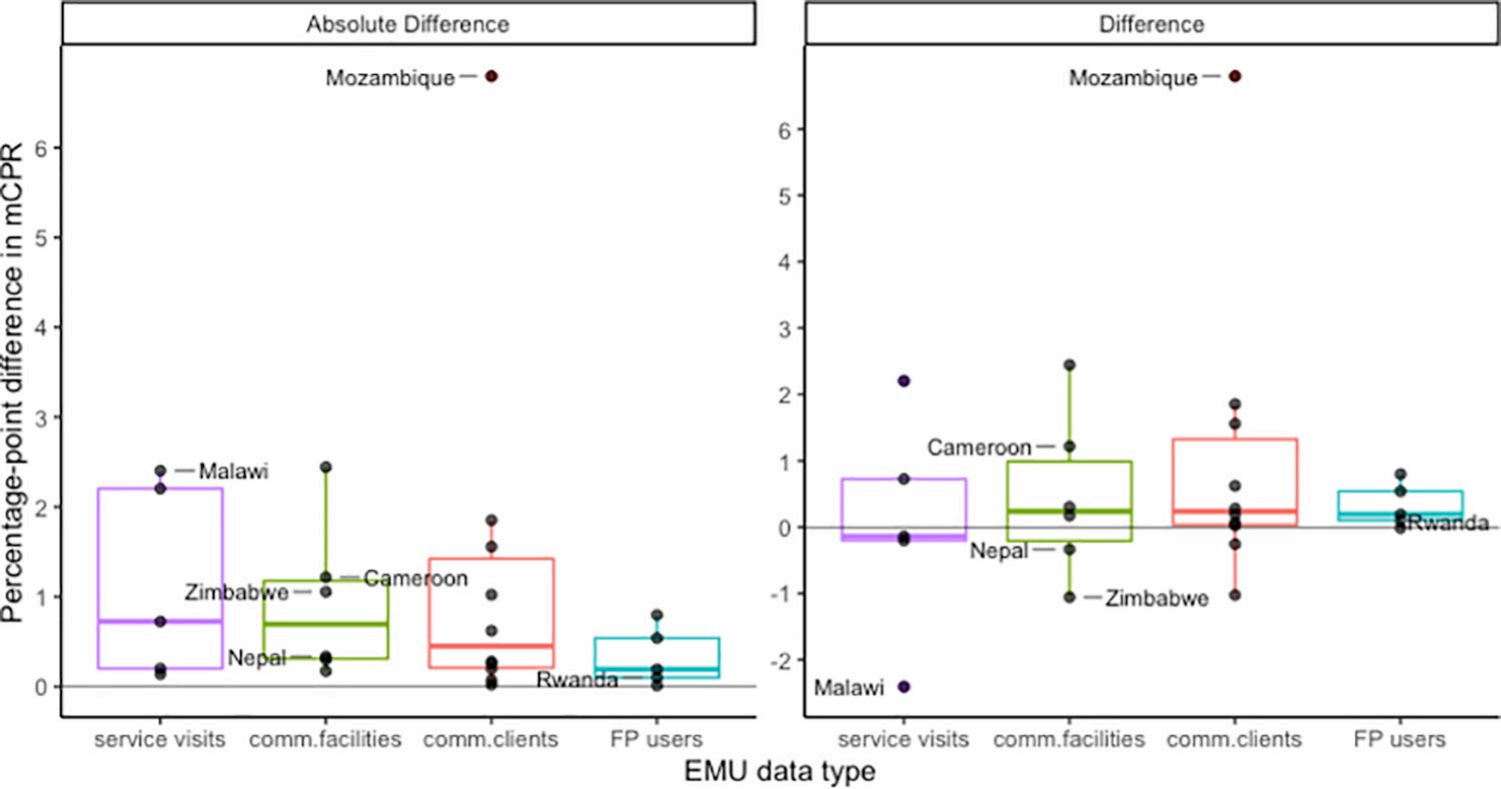
Differences in mCPR estimates including EMU vs excluding EMU. The plot shows boxplots of the absolute and raw differences between mCPR estimates obtained from a model that included an EMU modelling component and estimates obtained from a survey-only model.

We looked at the absolute difference in *fpemlocal* estimates of mCPR for the year 2020, using surveys only, compared to using surveys+EMUs (Fig 5). The use of the comm.clients EMU data type has the largest impact on average, showing a mean absolute difference of 1.3 percentage points. We found that the use of the FP users EMU data type had the smallest impact on point estimates of mCPR, with a mean absolute difference of less than half a percentage point. The mean absolute differences when using the service visits and comm.facilities data types were 1.14 and 0.92 percentage points respectively.

In terms of the impact on 2020 mCPR estimates for the 6 case study countries, we find that in Mozambique the inclusion of the comm.clients EMU data increases the 2020 estimate by 6.8 percentage points and in Cameroon the inclusion of comm.facilities increases the 2020 mCPR estimate by 1.2 percentage points (Fig 5). Conversely, the inclusion of service visits EMU data in Malawi decreases the 2020 mCPR estimate by 2.4 percentage points as compared to using survey-only informed estimates. Absolute differences of 1 percentage point or less are seen in Zimbabwe, Nepal and Rwanda.

#### Impact on projection uncertainty

**Fig 6:**
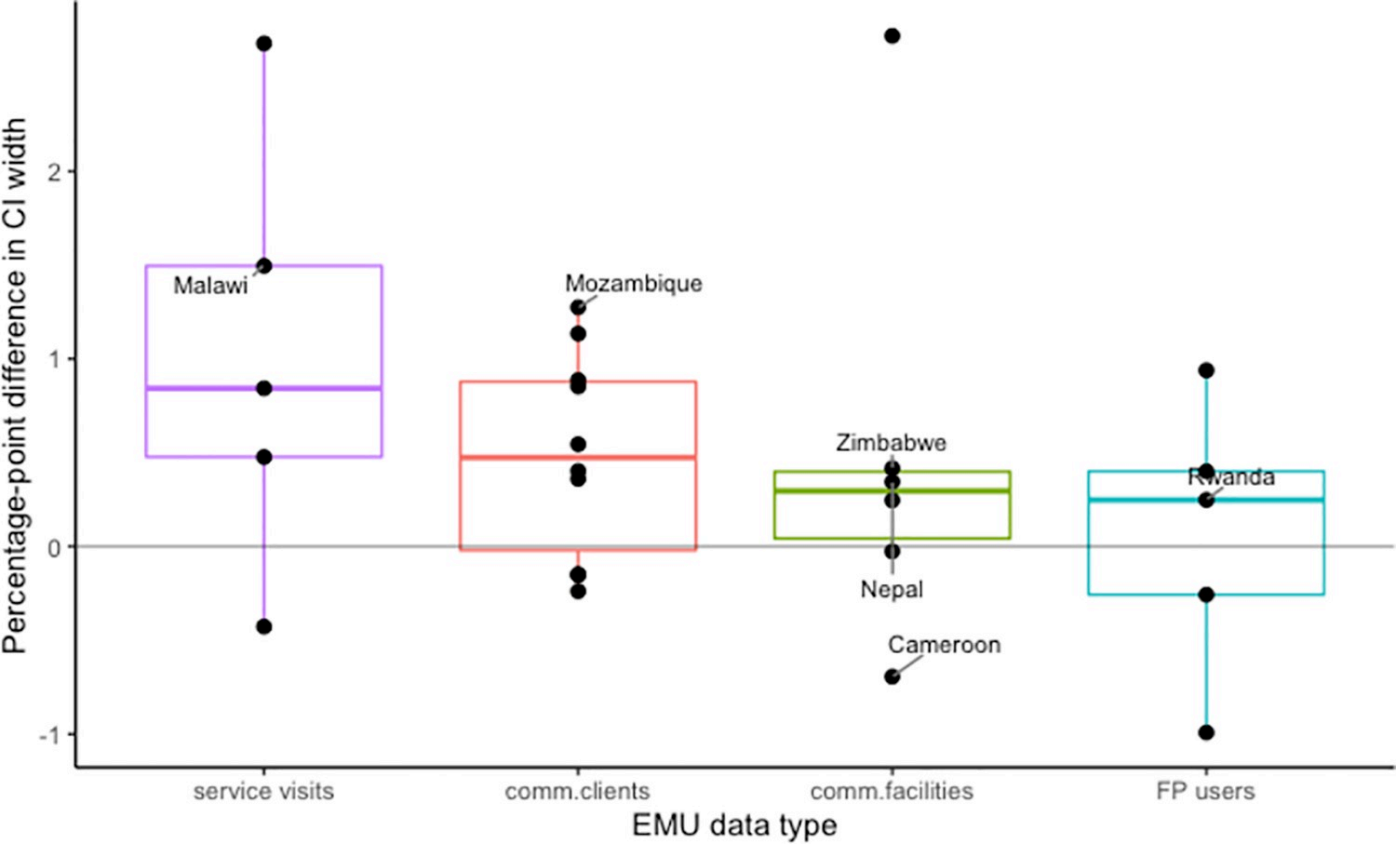
Differences in mCPR uncertainty including EMU vs excluding EMU. The plot shows boxplots of the differences between mCPR uncertainty interval widths from a model that included an EMU modelling component and a survey-only model.

Considering the width of the 95% uncertainty interval (UI) as the measure of uncertainty in the 2020 mCPR estimates, we found that the inclusion of the service visits EMU data type reduced the width of the UIs by 1 percentage point on average, compared to using surveys only (Fig 6). For countries that used comm.clients, comm.facilites and FP users, the average reduction in the width of the UIs was ~0.5, 0.5 and 0.1 percentage points respectively. The maximum reduction in the width of the UIs across all data types is 2.7 percentage points. The maximum increase is 0.9 percentage points.

For the 6 case study countries we see that the inclusion of EMUs reduced uncertainty for all countries except for Cameroon, which saw a 0.7 percentage point increase in the width of the UI. Malawi and Mozambique saw the largest reduction in the width of the UIs of 1.5 and 1.3 percentage points respectively.

## 5. Discussion

The assessment of family planning needs, including contraceptive use, has been reliant on data from large-scale surveys such as the Demographic and Health Surveys since the 1980s. However, reliance on surveys, which are generally carried out every 3–5 years, means that countries often lack recent data. This is particularly relevant when global FP initiatives such as FP2020 require monitoring of FP indicators on an annual basis [4]. In the absence of recent survey data, countries are forced to rely on model-based projections to gain insight into the current state of modern contraceptive use and other key indicators. Model estimates and projections come with an inherent uncertainty, with projections having larger uncertainty due to a lack of data. FP service statistics provide an opportunity for FP models to become less reliant on projections for assessing recent trends. Typically, if contraceptive use is changing at a rate that is consistent with recent past trends then this is likely to be reflected in the service statistics and the inclusion of EMUs in FPEM will only have a small impact on estimates and projections. Conversely, if more dramatic changes in contractive use are happening, but there are no recent surveys to inform FPEM estimates, then the service statistics, which will be tracking these changes in real time will fill the knowledge gap (e.g., Fig 4 - Mozambique). Therefore, the inclusion of information from service statistics in FPEM allows us to produce more informed data-driven estimates of key FP indicators up to the current year and into the future. This will be of particular importance for monitoring changes in contraceptive use at the subnational level, where data are even more sparse and come with a larger uncertainty compared to the national level.

While data derived from service statistics have a number of strengths they also have a number of weaknesses that need to be carefully considered. These include that they are (1) prone to reporting and processing errors and are (2) generally biased with respect to the population-level indicators they are trying to measure [11]. The question becomes: Can we make use of service statistics in spite of their shortcomings? Studies have shown that the trends in EMU observations derived from service statistics track with survey-based trends in mCPR [11]. Based on this information, we have developed an approach to include EMU data in the *fpemlocal* R package to allow changes in EMU to inform estimates and projections of family planning indicators, including mCPR. Within *fpemlocal*, annual changes in EMU are assumed to vary around annual changes in mCPR. We estimated the magnitude of the variation through exploring differences between changes in EMU and changes in mCPR that have been derived independently of EMU. Validations showed that the addition of the EMU modelling component in *fpemlocal* reduced the RMSE by about 7% and that coverage remained on-par with the survey-only model.

We found that overall the inclusion of the EMU modelling component in *fpemlocal* had an impact on the 2020 point estimates of mCPR compared to using a survey only model. The effect on point estimates is most apparent for the comm.clients EMU data type, where the absolute change in mCPR between the two models was 1.3 percentage points on average. The largest change overall of 6.8 percentage points was seen in Mozambique. With respect to uncertainty, the inclusion of EMU resulted in differences in the width of the UIs for mCPR in 2020 ranging from an increase of ~1 percentage point to a decrease of ~3 percentage points. On average, the inclusion of EMU showed small decreases (between 0.5 and 1 percentage points) in the width of the UIs across all data types, with the exception of FP Users which showed an average decrease of ~0.

With the addition of the EMU data model component to FPEM, we have acknowledged and accommodated the drive towards the use of family planning service statistics in recent years by the global FP2020 initiative. We foresee future work in this area focusing on investigating if/how improvements can be made in the derivation of EMUs from service statistics and understanding the settings in which EMUs do or do not track well with mCPR in order to reduce bias and/or variance. For now, we highlight that a key advantage of pivoting to the use of data derived from service statistics in FPEM is that they provide a higher spatial and temporal resolution than surveys. The high geographic detail can be obtained even down to the service delivery point level. In addition there has been numerous efforts in recent years to improve the tools for routine data collection and service statistics are available frequently—usually monthly, and potentially in real time [11, 21]. This could be of particular benefit in a subnational setting where the EMUs could carry relatively more weight given that subnational survey estimates tend to have a larger associated uncertainty compared to the national level. A second major advantage of using service statistics is they are routinely collected through health information systems and therefore they are extremely cost effective in comparison to the high cost of national surveys [22]. Here, we have demonstrated how service statistics can be used in practice, to inform estimates of key FP indicators. We provide freely available tools to enable in-country usage for improved tracking of progress towards country-specific FP targets and goals.
